# Performance, Health, and Psychological Challenges Faced by Students of Physical Education in Online Learning during COVID-19 Epidemic: A Qualitative Study in China

**DOI:** 10.3390/healthcare9081030

**Published:** 2021-08-11

**Authors:** Rizwan Ahmed Laar, Muhammad Azeem Ashraf, Jin Ning, Peigang Ji, Ping Fang, Tianran Yu, Muhammad Naeem Khan

**Affiliations:** 1 College of Physical Education, Hubei Normal University, Huangshi 435002, China; rizwan_laar@yahoo.com; 2 Research Institute of Educational Science, Hunan University, Changsha 410082, China; jinning@hnu.edu.cn; 3 School of Sports Science and Physical Education, Nanjing Normal University, Nanjing 210023, China; jpg@njnu.edu.com (P.J.); 191501001@njnu.edu.com (P.F.); 4 School of Health and Physical Education, Huaihua University, Huaihua 418099, China; ytr@hhtc.edu.cn; 5 School of Social and Behavioral Sciences, Nanjing University, Nanjing 210023, China; naeem@smail.nju.edu.cn

**Keywords:** COVID-19, quarantine, online classes, health issues, physical education students, weight gain, poor performance, psychological issues

## Abstract

The spread of COVID-19 has led students to take classes online (rather than face-to-face) worldwide, including in China. For this study, we conducted qualitative focus group discussions to identify the experience of and difficulties faced by online physical education students in higher education taking online classes, including the impact on their physical activity performance, as well as some health problems they face while taking online classes during the quarantine period, such as weight gain, depression, and anxiety. Finally, utilizing Mayer’s learning model as a conceptual framework, we propose a method that addresses how to effectively manage an optimal future learning system for physical education students, both during and after the COVID-19 outbreak situation. During the isolation period of COVID-19, the required equipment for the participants was rarely available at home while attending the online classes, which inevitably reduced the number of physical education units that could be conveyed. This resulted in a transfer of attention from competition to underperformance, weight gain, and adverse psychological conditions. We conclude that it is important to review and systematize the methods of online physical education learning, particularly highlighting the cultural and educational characteristics of different countries, and to investigate the effectiveness of online physical education as a whole.

## 1. Introduction

The spread of COVID-19 has led to the interruption of more than 850 million students and has disturbed the currently available teaching strategies of educational institutions around the world. According to United Nations and UNESCO, the epidemic has caused significant damage to the education system, including the closure of schools, leading to huge pressure being placed on parents, students, educators, educational institutions, and governments to ensure the continuity of learning. This is expressly true, as many teachers and students have little to no online learning experience [1]. The outbreak of COVID-19, which began at the end of 2019, has rapidly changed the national emergency policy in China. Soon after, most countries began to provide online teaching to students through different online applications. In order to promote online education and the temporary ceasing of normal teaching orders, on 6 February 2020, the Ministry of Education of the People’s Republic of China started to take online teaching methods into consideration [2]. In addition, the Chinese government has launched various emergency measures, including social alienation, such as locking down cities and closing schools [3,4,5]. An emergency policy was initiated by the Ministry of education, called “Suspending Classes Without Stopping Learning,” in order to turn teaching activities into online teaching methods when the schools are closed. It is worth noting that the policy of “Suspending Classes Without Stopping Learning” does not completely follow the standard policy-making process, such that the gains and losses in the implementation process of e-learning and -teaching deserve serious consideration and research [6].

During the COVID-19 pandemic in 2020, online physical education classes have been run almost all over the world, which is a new experience for both students and teachers. Different from the general knowledge focus of subjects, physical education focuses on sports activities, which is a demonstrative field. All participants are anxious about how to communicate sports activities in online physical education classes, as well as how to make online physical education a meaningful educational activity. In a study on physical activity restrictions, Kim et al. discussed that different health-oriented physical activities should be introduced in online classes, as most participants, despite their age and gender, have health issues [7]. According to the World Health Organization (WHO), the first sign of community health is physical activity [8,9]. Obviously, physical activity plays a crucial role in the physical and social development of an individual. However, many negative effects of physical inactivity on health-related problems have been widely highlighted in various scientific studies. Non-communicable diseases, such as weight gain, and chronic health conditions are mainly related to a lack of exercise [10,11]. Notably, in online physical education classes, students have the least chance of participate in sports activities when they are at home with limited sporting facilities. During the epidemic, due to the rapid transition from higher education to online teaching, the well-being of physical education staff and students has been affected [12]. It is hard to convey the value and main objectives of sports in online physical education classes. These values and objectives include developing sports protocols through participation in sporting activities, promoting community awareness through sporting activities with family and friends, and keeping healthy by participating in physical activities. It is worth noting that students who take online physical education classes often do not have adequate opportunities to participate in sports activities effectively, and their access to sports venues and other supplies, equipment, and facilities become limited, for obvious reasons. Therefore, the participants of online physical education classes use limited sports equipment at home, which inevitably decreases the number of sports units that can be learned. This results a shift in focus from competition, which is a main part of physical education, to underperformance, health, and physical activity challenges in online physical education classes. Proactive actions are needed during the COVID-19 pandemic, in order to support the health of students and to address other sport performance-related challenges. This is particularly important to reduce the associated disturbance to education and the transition to a graduate career [13]. Hence, long-term and flexible plans must be considered, in order to cope with the impact of the pandemic and other possible disruptions to education. On the other hand, these programs need to take into account the attitudes, outcomes, suggestions, and concerns of teachers and learners, based on their experience in the first wave of COVID-19 [6,14,15], which makes the current study and other similar studies important.

Notably, physical education mainly depends on sports activities, which is obviously different from other disciplines such as general knowledge or history. Therefore, online physical education teaching requires distinct preparation and procedures in order to better exchange and practice the values of physical education; otherwise, online learning may produce adverse side effects, especially for students majoring in physical education, including low performance (due to a lack of or no practice), and some medical conditions such as weight gain, anxiety, and so on [16]. Therefore, it is necessary to check whether online physical education classes are being conducted to appropriately convey the values of physical education. However, previous studies on the effectiveness and efficiency of online physical education classes are limited [17,18]. Most of the existing literature has only observed the effectiveness of college classes in a limited scope; to the best of our knowledge, there has been no research investigating the difficulties in practicing related sports and other challenges (as mentioned earlier) of higher education physical education students during online classes. Therefore, it is necessary to highlight the existing practices and challenges for online physical education classes. The current study focuses on various difficulties in Higher Education online physical education classes, such as the impact on performance in any sport or physical activity and some medical issues (e.g., weight gain and depression). Finally, taking Mayer’s learning model as the conceptual framework of the current research (discussed in detail in the “Materials and Methods” section), we propose a method for effectively managing future learning systems for physical education students, both during and after the epidemic. The results of this research can be used as a basis for the future revitalization of physical education.

## 2. Literature Review

The worldwide COVID-19 epidemic has led to the closure of stadiums, gyms, swimming pools, physiotherapy centers, dance and fitness studios, playgrounds, and parks. As a result, many individuals have become unable to actively participate in their regular individual or group sporting activities outside their home. In this case, most people experience less physical activity, longer screen time, irregular sleeping habits, and worse diet, which lead to weight gain and loss of health. Low-income families are particularly susceptible to the negative impact of lockdown rules, as their accommodation conditions are often sub-standard and their living space is narrower, such that it is difficult for them to carry out physical exercise. This condition has led to a large-scale transformation, which has impacted the educational system in that countries all over the world quickly closed down in-person classrooms and turned to teaching in different virtual environments at the time of the COVID-19 epidemic [1].

Among the many significant topics involved in previous studies are the differences between emergency and quality online learning, the value of different educational methods, and giving priority to the health of students, rather than their academic performance, as well as the main challenges faced by students and suggestions to overcome them [1]. Many studies have warned against confusing well-planned online learning, which demands long-term planning, instructional design, and special instructional infrastructure, as well as the rapid and temporary conversion to online learning, in order to maintain teaching under the special conditions of COVID-19 [15,19,20,21]. In this case, it has been suggested that confusing high-quality online learning with emergency online learning may impact the performance of teachers and students in the context of sports teaching, and may cause some health complications (e.g., weight gain and anxiety), due to a lack of physical activities during online physical education classes [15,19,22]. Another significant topic discussed in the concerned studies is that, due to the limited resources available during the period of COVID-19 online physical education classes, students have been faced with the problem of lack of practice, and the issue of how to ensure the health of students, as well as their access to a fair and inclusive learning environment, have received significant attention [21,23,24,25,26,27]. On the other hand, various studies have emphasized that educational institutions need to give priority to the physical and mental health of students and educators, rather than teaching courses [28,29,30].

Another group of studies has highlighted the attitudes of teachers and/or students toward switching to online education in COVID-19. These studies show that, on the whole, students have adapted well to the new learning experience. However, most studies have emphasized that there exists a significant correlation between the socio-economic condition of the participants and their attitude, where the privileged are more satisfied [19,20,31,32]. In addition, some of these studies have focused on the various barriers that may hinder the effective provision of online education, including the fact that most institutions, staff, and students are not prepared to participate in large-scale and urgent online courses [6,19,33]; the economic, digital, social, and gender differences among members that directly affect the availability of online teaching infrastructure and the availability of technology and internet facilities [6,31,32]; and insufficient psychological, academic, and social support for students [19,31]. However, the current research mainly focuses on the adverse effects of an unfavorable home environment on the performance of many physical education students in sports events, as well as some health (e.g., weight gain) and psychological (e.g., anxiety) problems they face during online physical education classes. These barriers are expected to increase the sensitivity of participants to psychological stress, and require a caring and empathic approach to tackle [19,21,28].

## 3. Materials and Methods

This study adopts a qualitative case study approach, with the use of phenomenological procedures to collect and analyze data [34]. We highlight the difficulties of holding online physical education classes, as discussed by the participants, revealed their effective operation and the challenges faced therein, and observed the data through thematic analysis [35]. This study is part of a large project funded by National Natural Science Foundation of China, and it followed all ethical considerations. All participants gave their informed consent prior to their participation in the study. The study was conducted in accordance with the Declaration of Helsinki, and the protocol was approved by the Ethics Committee of the School of Sports Science and Physical Education Nanjing Normal University. We completely followed the relevant ethical considerations.

### 3.1. Participants

In order to assess the challenges faced by students during online physical education classes in China, 56 students, enrolled in physical education programs at nine different higher education institutions, participated in this study. Teachers and class leaders of physical education students enrolled in bachelors, masters, and PhD programs were contacted to help the researchers to recruit participants. The teachers and class leaders acted as connectors, as they had direct contact with the students. An invitation letter was sent to students, with the help of the class leaders and teachers, in June 2020. The letter detailed the aims of the study, and the recipients were informed that their participation in the study was voluntary in nature. A reminder invitation letter was sent to the participants in July 2020. In total, 64 students responded to the invitation; however, 56 students agreed to participate in the study, and were assigned anonymous names from P1 to P56.

### 3.2. Data Collection

The data included six focus group discussions using the Tencent Meeting application, which has been widely used in China for conducting meetings and classes during COVID-19, and has an option to record the virtual sessions [2]. The focus group discussions were conducted in the Chinese language, and examined the experiences of the participants during online classes. The focus group discussions were centered on the discussion guide developed by two of the authors (R.A.L. and M.A.A.). We consulted experts in this field to develop the guidelines for focus group discussions in the study (see Table 1).

**Figure 1:**
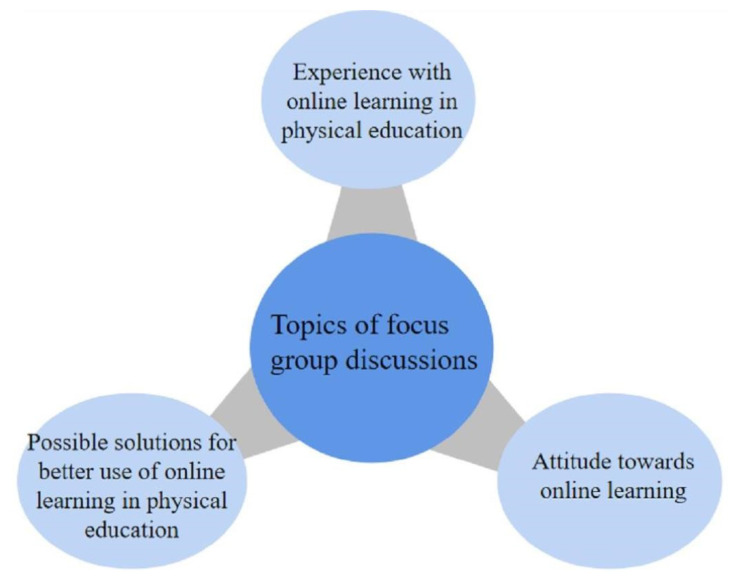
Topics for focus group discussions.

The focus group guidelines were first approved by all authors, and a pre-test was conducted on six students before the formal discussions. In this pre-test, students were asked to check the guidelines, discussion questions, process, and discussion settings. The pre-test was considered effective, as it helped the researchers to check the appropriateness of discussion questions, and confirmed the difficulty of answering questions in an online setting. The discussions allowed participants to share as many features as possible. The focus group discussions were organized from August to December 2020, and each session lasted 50–75 min. The discussions were mainly focused on the experience of students in online physical education classes, including the challenges that were encountered and overcome in the online physical education classes (see also [16]). Figure 1 presents the guided topics used for focus group sessions in this study.

Each focus group discussion started with an introduction, and continued with questions about the experiences of participants with online physical education classes, the challenges they faced, distinctions between online and on-campus learning, and suggestions for making online teaching more interesting for students and teachers in physical education. All participants in each focus group took part in a live discussion session by logging into Tencent Meeting. Three moderators were present during each session-one moderator was responsible for the smooth progression of each session, the second moderator had the duty of making sure that all the topics in questions were included, and the third moderator was responsible for taking notes, recording the session, and time keeping. These triple-moderator focus groups were found to be very productive in data collection.

The focus group sessions were composed of 11 open-ended discussion questions for the participants, which facilitated collaboration, interpretation, and reflection within a seminar format. Table 2 presents the questions that guided the focus group sessions. The questionnaire was designed in accordance with the topics of the research presented in Figure 1. At first, opening questions were focused on participants’ previous experience with online learning, while exploratory questions were aimed at participants’ present experience with online learning. Transition and key questions were aimed at learning participants’ attitude towards online learning. The closing questions focused on participants’ suggestions for effective future online learning in physical education.

All focus group discussions were recorded, transcribed into MS Word, and then translated into English for data analysis purposes [35]. A Chinese researcher who is fluent in English worked as a moderator during the focus group discussions. In addition, other authors were also present during the focus group discussions, who are fluent in both English and Chinese (holding a Chinese language HSK certificate). A Chinese researcher transcribed and translated the data, which were checked by other authors who are fluent in English and Chinese. Key rich information obtained by the interviews/discussions can be explained through the work of Rosenthal and Fischer-Rosenthal [35,36] who state that in order to explain and understand the participants’ comments about their previous experiences, it is very important to explain some parts of their current life and future outlook (p. 259).

### 3.3. Data Analysis and Research Authenticity

The collected data were analyzed using a thematic analysis approach [37,38]. First, the researchers read the material repeatedly, in order to understand the whole process and the true meaning. In order to determine the overall structure, classification and grouping of topics were conducted and, through the analysis of technical, reflective, and explanatory writing, the relationships between the basic elements of the results were classified. Based on the objectives of the current study, initial codes were created. The process of grouping of the codes into main themes followed and, after refinement of the themes, a report was produced (see Table 3). The categorization after coding was classified into themes, such as challenges in running online physical education classes (sub-themes: impact on performance in sport or physical activity, weight gain, and psychological issues) and suggestions for running more efficient online physical education classes (mentioned below in more detail). Finally, integration, an iterative process of reinterpretation, and amendments were conducted, in order to confirm that the designed themes properly reflected the aim of the current research. In order to improve the effectiveness of the research and to test the consistency of the research results, the triangulation approach was used to cross-verify the collected data and the researcher’s notes from different angles. The participants reviewed the results in order to ensure that their meaning was correctly expressed. Through the continuous feedback of two qualitative research experts (chairman, vice-chairman, and two related professors at the School of Sports Science and Physical Education, Nanjing Normal University) on the whole research process, the quality of the research was ensured.

### 3.4. Conceptual Framework

**Figure 2:**
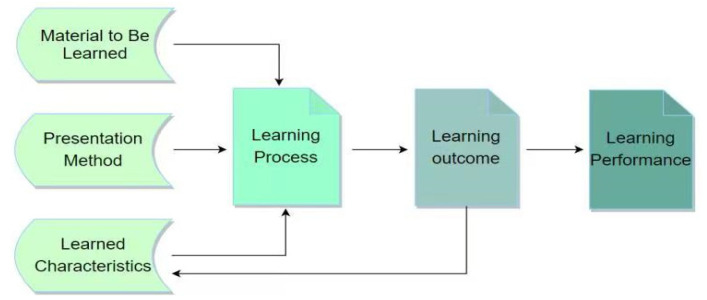
Mayer’s Components of effective Learning (Mayer, 1989; p. 45) (Authors’ illustration).

The theoretical framework of the current research is based on the learning model proposed by the educational psychologist Richard Mayer [39]. There are three dimensions in the model; namely, Materials to be Learned, Presentation Method, and Learning Strategies or characteristics (see Figure 2). Each dimension represents the choices made by the course designer while designing a course. Therefore, courses should be designed accordingly in order to have better learning results for the students. According to Mayer (1989), the learning outcome or performance is significantly connected with three dimensions of the model, which are universal for face-to-face or online classes (see, e.g., [39,40]). The material to be learned is an important element of the model. “Material to be Learned” refers to the skills and conceptual ideas that are offered in the course. Meanwhile, the technique used to present the materials to participants is called the presentation method. The “Presentation Method” is an element in the framework, in which curriculum designers decide where, how, and when to deliver the materials. In addition, learning strategies or characteristics point out a comprehensive direction for course designers in delivering and developing course materials. In other words, the “learning Strategy or Characteristics” are used to reply to the question of “Why” the material will be delivered, using the delivery methods the course designer has chosen. In order to achieve effective and efficient learning outcomes or performance, these three aspects are significantly useful while designing either face-to-face or online courses [39,40].

## 4. Results

Among the participants, 34 participants were male and 22 were female, 21 participants were enrolled in bachelor’s programs, 20 were enrolled in Master’s programs, and 15 were enrolled in PhD programs in physical education. Table 4 shows the background information of the research participants.

The thematic analysis of focus group discussions resulted in three major themes and several sub-themes. The major themes included: (1) knowledge of online learning; (2) challenges in running online physical education classes; and (3) suggestions for running more efficient online physical education classes. Table 5 shows the themes and sub-themes that originated from the focus group discussions. All themes and sub-themes are outlined below, with related quotes from the participants.

### 4.1. Knowledge about Online Learning

The first theme that emerged from focus groups was the knowledge of participants about online learning and the role of online learning in physical education. This theme consisted of three sub-themes: (1) familiarity with online learning; (2) awareness of course content; and (3) difficulties in course content understanding.

#### 4.1.1. Familiarity of Online Learning

Among our participants, most participants were familiar with online learning; however, very few (eight participants) had experience with online learning prior to the pandemic. Among these experienced participants, all had used online learning for other subjects, such as English. All participants had never taken any physical education class through online learning, and neither had they witnessed whether online learning had ever been used in their departments. However, many participants (25 participants) had used digital tools, such as recorded videos of physical trainers and sports persons, to improve their performance.

“I have taken few English courses online, to improve my spoken English. However, I have never taken any physical education course online prior to pandemic.”

(Participant in focus group 2.)

“I know many friends who took their courses online, mainly through MOOC, but I have never heard if there is any physical education course.”

(Another participant from focus group 2.)

“I have used online tools to improve my performance in physical activity. May major is badminton, and I often watch recordings of top players to improve my performance and strategy in game.”

(Participant from focus group 3.)

#### 4.1.2. Awareness of Course Contents

The second sub-theme was focused on participants’ understanding of course content. The participants showed different degrees of understanding of the course content in online learning mode, wherein a majority of participants agreed to the fact that online learning is beneficial to some disciplines; however, its role in physical education is limited.

“I think online teaching is effective for theoretical subjects such as history of sports, but it is less effective in teaching courses that require physical activity.”

(Participant from focus group 3.)

“Some online classes were very helpful, such as sports nutrition, and I really enjoyed learning them. The best part of it was that I could record the lectures, and learn it again whenever I need.”

(Participant from focus group 5.)

“Online lectures offered me an opportunity to pay more attention to the contents of the course, and listen to each lecture carefully.”

(Participant from focus group 6.)

Similar to these participants, the majority of other participants described online learning as an opportunity to focus more on lectures. They mentioned that recording lectures and watching them again helped them to better understand the lecture contents.

#### 4.1.3. Difficulties in Course Content Understanding

The third sub-theme was concerned with the difficulties that the participants faced in understanding the content of the online courses. The participants showed great variation in terms of their desire to receive online content, the learning needs of different types of learners, and the different levels of satisfaction with online learning content. Some examples are given below:

“It was difficult for me to understand few online classes, mostly those that involved physical activity.”

(Participant from focus group 1.)

“I think teachers’ presence in classroom is very important; it gives me feelings of being involved in real learning session. I missed it in online classes.”

(Participant from focus group 4.)

“Learning on-campus is very important for me, because it allows me to discuss study related issues with classmates and friends, and I could communicate easily with teachers.”

(Participant from focus group 4.)

“In my opinion, online lectures are only useful during pandemic, and there is no use of online teaching in real practice. Because, it is difficult for students like me to engage in productive discussions during online lectures, which is easy in on-campus classes.”

(Participant from focus group 6.)

“For me, the learning environment is everything. I cannot learn if I feel learning environment is distracted. Online learning did not give me feelings of learning environment, only on-campus classes provides true learning environments. And we can talk to other classmates in person, we can practice together in playgrounds, and we can hang out together.”

(Another participant from focus group 6.)

### 4.2. Challenging in Running Online Physical Education Classes

The following section focuses on the participants who perceived difficulties during their first time attending online physical education classes, such as its impact on their performance in sport, limited environmental opportunities for physical activity that did not properly transfer the core value of physical education, weight gain due to a lack of physical activities during online physical education classes, and psychological issues (e.g., depression, inferiority, or poor self-esteem and anxiety).

#### 4.2.1. Impact on Performance in Sport or Physical Activity

Some of the students in the study were strongly disturbed by lack of availability of resources for training or practice at home during online classes.

“Physical education classes are not like other subjects like Psychology or Education, in which you rarely have to do something with physical practices or experiments. It is very difficult, for me and all other physical education students to have all required facilities and equipment at home while online classes, I personally feel my performance in any athletic activity is decreasing day by day due to lack of practice.”

(Participant from focus group 5.)

“In online learning, students do not have a proper chance to receive feedback by seeing their own or other students’ actions. This is in contrast to face-to-face physical education classes where participants can instantly get response on their motor skills. This is one of the major drawback of online physical education classes.”

(Participant from focus group 6.)

On the other hand, three focus groups out of six responded relatively similarly, with statements from some of them given below:

“During the online learning, I had always doubts about whether the values and core purposes of physical education that we supposed to get were being learned well, while knowing the fact that students had to practice by themselves with whatever limited environment and facilities or equipment they have at home.”

(Participant from focus group 1.)

“During online classes some of our teachers had no option but to design monotonous classes, for example: juggling and “challenging” stay-at-home challenges that could be done in participants’ (students) houses because of not having access to required equipment or ground, which had caused lack of performance in sporting activities in long term.”

(Participant from focus group 4.)

A participant from focus group 5, on behalf of all physical education students who are taking online classes, argued that “students cannot adjust self-activities by viewing a short video of themselves during online learning. A quick response is necessary to encourage students to learn or modify and reinforce their athletic performance. In addition, insufficient interaction between the students and teacher during online classes made it more hard to express the value and main object of physical education which leads to poor performance.”

#### 4.2.2. Weight Gain

In the current study, most of the physical education students faced health condition issues (mainly weight gain) due to physical inactivity during online courses as a result of the COVID-19 epidemic. According to a participant from focus group 3, China faces growing problems with weight gain and psychological problems (further discussed below), especially under the pandemic (COVID-19) conditions; he further added that:

“Chinese peoples are on top of fifth position in the world ranking of overweight or obese people and this proportion seems to have increased in confinement and online courses, especially among physical education students. Weight gain is can be cause of Western eating habits, automobile transportation and sedentary lifestyle. Usually (in face-to-face class) we (students) indulge in sports activities for 4 to 5 h every day, but in online class, due to the limited resources at home, our opportunities to participate in such activities are very limited.”

(Participant from group 3.)

The story of one of participant from focus group 3 is fascinating. She narrated that:

“Pandemic just started around the time of Chinese new year 2019, normally we make a lot of traditional food and variety of dishes to eat and celebrate with family members. Eating habits plus and less physical activity chances during online classes results in increase of weight not only physical education students but most of all Chinese peoples. There is a pressing need to reduce weight gain in China’s growing population.”

“The overweight rate and fertility rate have increased consistently, during the lockdown of COVID-19. My wife got pregnant with 2nd child without planning during pandemic. In results both got weight gain. It was really hard to not go outside and even for classes we need to be online and can’t leave houses.”

(Participant from focus group 1.)

In the context of focus group discussions with the aforementioned participants, physical training or practice becomes a significant factor for the understanding of the values and core objectives of physical education, as well as avoiding overweightness. In addition, it is clear from the results that online physical education classes contribute significantly to weight gain. Notably, researchers have observed that the global goal set by the World Health Organization (i.e., not to surpass the 2010 limit of weight gain by 2025) is nearly impossible to reach, especially considering COVID-19 [41]. This experience recommends that efforts to transfer the values and purposes of physical education and the controlling of increasing weight gain in China should be tackled as a further solution, after the technical skills for different sports have been studied [17].

#### 4.2.3. Psychological Issues

One participant from focus group 4 said that the online classes and quarantines have led to mental pressure for most residents. She further continued:

“During the online classes in COVID-19 pandemic physical education students were not only facing the problem of lack of physical trainings or activities but they were also suffering from some mental conditions. Day went after, we just lost our hope to continue our normal (face-to-face) classes as we used to attain before COVID-19.”

According to a participant from focus group 5,

“Two things kept disturbing psychologically during the time of COVID-19 pandemic. First, we were stuck at home during quarantine, and second we did not have any chance for exercise. We know, physical activity keeps a body fit, and psychologically strong. It is almost impossible to gain these benefits during online classes.”

### 4.3. Suggestions for Running More Efficient Online Physical Education Classes

Online physical education classes must convey the value and purpose of physical activity as a significant component of health [42]. However, before teaching students the values and purpose of sports, teachers should encourage students to vigorously participate in the online classes at the same time and, thus, to pay attention to the concepts of physical education. Online physical education classes should guide students to develop their future sports plan and self-directed ability subjectively. Although the courses offered via the internet have no time and space restrictions, and almost everyone can access them, if students inactively and irresponsibly participate in physical training or activities, such courses will be inefficient and ineffective. In other words, the attitude of students towards autonomous learning is an important factor which influences the effective running of online physical education classes. Therefore, it is important that teachers develop education plans for online classes to help students form a good learning attitude. In addition, they should stimulate students to participate in sports activities, helping to transfer the value and main purpose of physical education [43].

Participant from focus group 3: “At the time of teachers training, the main issue was no matter how much effort is made by the teachers to held a good online class, it will be worthless if the students participate inactivity and start doing practice at home with whatever limited resources they have.”

Participant from focus group 6: “In online lectures, teachers must give assignments according to available facilities at home. It would be impossible to follow if there is no facility at home, even students are actively participating in online classes and doing required trainings. In addition, indirect experiences of physical activity based on direct experience should convey the value and main purpose of physical education through online classes.”

“Before discussing the importance of physical education, it’s better to use different educational means and teaching materials to let students actively participate in online classes with the attitude of autonomous learning. We should make full use of all kinds of video images, debates, discussions and reports that are not fully used in the current physical education classes, and strive to convey the value and main purpose of physical education.”

(Participant from focus group 6.)

Online physical education classes are obviously not the same as traditional (face-to-face) physical education classes. The participants had different suggestions on how to effectively set up online physical education classes. Such changes are very important for the development and application of group work which encourages students to participate, in order to overcome the shortcomings of online physical education classes [44]. In the future, new assignments need to be developed in order to enable teachers to identify the learning status of individual students, just as participants adopt various educational plans to increase the values and purposes of a class.

## 5. Discussion

In the context of COVID-19, the current study examined the experience of Chinese students with online physical education classes and puts forward effective operational learning and teaching strategies for future online physical education classes. The notion of online classes is not new in the Chinese higher education setting; however, its role in teaching physical education classes is limited. In the discussions, participants described different factors that influenced their behavior in experiencing online learning in physical education. Participants who supported online learning were inspired by the advantages of acquiring knowledge of the content in a more effective way compared to campus learning. As with other previous studies [16,20,28], the participants of the current study also employed modern learning technologies to enhance their learning, as well as to improve their practices and strategies. In addition, online learning assisted participants by empowering them in controlling their learning needs, and guided them to conduct self-controlled learning [45]. It has been confirmed that physical exercise can lead to benefits in many aspects, including the improvement of mental health, the prevention and treatment of chronic diseases, and so on. Online physical education classes mostly deprive participants of the physical exercises they usually participate in [46]. Isolation during COVID-19 has had adverse effects on human health, which may result in depression, inferiority, or poor self-esteem and anxiety. On the other hand, sports activities do not always need to be at the center of establishing sports value in actual (face-to-face) classes: Park et al. reported that, in online physical education classes, it is sometimes important to establish sports values based on various types of materials and audio-visual equipment and activities, in order to support the positive health behaviors of students [47]. It is necessary to develop methods to connect emotional regions, as well as to expand cognitive and defining areas, which may be the advantages of online physical education.

The way in which a few of the students felt psychological conditions due to a lack of physical activity during online physical education classes and quarantine supported much of the previous literature, especially the assertion, regarding COVID-19, that physical inactivity during online physical education classes is “problematic” [16]. On the other hand, exercise and physical activity researchers and public health practitioners have labeled online courses as one of the main reasons for poor psychological conditions [48]. According to the results, it is obvious that the students experienced plenty of hurdles to the practice of physical activity during online physical education classes; for example, according to a participant from focus group 6, in online classes, students can not directly receive a response to their motor skills, which may cause a lack in terms of performance and ability during a competition or normal exercises [7,16]. Keeping in mind the results of the current study, it is clear that most of the physical education students were not satisfied by taking online physical education classes and strongly complained that online physical education courses significantly impacted on their performance due to inconvenience, limited facilities available at home, and having no access to the required sport courts or grounds. This experience suggests that the interaction between students and teachers, equipment, and required facilities are the most important factors for maintaining good performance and to achieve the real purpose of the physical education field [49,50]. However, many participants also explained difficulties that they faced in understanding the online course content. These students found online learning difficult to master, in terms of the required knowledge, as some factors were considered ineffective for learning. The challenges that the physical education students mainly faced during online courses included the monotony caused by the inadequate environmental conditions and insufficient practical activities, which ultimately reduced the effect of the physical education classes. The results of the current study are in agreement with those of previous studies [7,16,49], which concluded that online physical education classes have a tremendous impact on the performance of students in any sports competition or in daily practices.

Therefore, it is important to explore the value and main purpose of physical education teaching and practical training in online classes (see [14,31]). Secondly, physical education teachers in China lack the professional knowledge which can be used in the development of online content, so they simply adopt a trial-and-error method for better learning. In order to solve these problems, we expect that, due to the outbreak of COVID-19, the effective content will develop in different directions. Thirdly, according to the evaluation policy recommended by the Ministry of education of China, student evaluation is very limited. On the other hand, due to the online nature of the class, it is impossible to carry out systematic evaluation. As most colleges and universities in China are still implementing preventive measures, and most colleges and universities are conducting online courses, it may be necessary to build a fresh assessment approach that can effectively operate in online classes. In addition, in order to plan an effective online physical education curriculum, we need to combine online sports characteristics with strategic learning methods, in order to help teachers to convey the value of sports to students. In the transmission of physical education values, which is the main object of physical education in China, solving the problem of a lack in the psychodynamic and affective domains will serve to enhance the efficiency of online physical education classes. Fourth, physical education teachers need to design future physical education teaching methods and obtain skilled practical knowledge by sharing online physical education content. This kind of cooperation between physical education teachers is essential, and must incorporate the professional knowledge of the China Physical Education Research Association. Finally, it is important for students to strive to actively participate in online physical education classes and record the process through the discussion of evaluation methods and patterns that are suitable for online physical education classes, as well as to try to keep themselves busy (especially during online classes in quarantine) with physical exercises using all of their available resources, in order to continue to avoid weight gain and adverse psychological conditions.

The participants of the current study had no significant experience with information or communication technology when faced with the emergency outbreak of COVID-19, but they still actively participated in online physical education classes, played the role of Chinese representatives as best as they could, and made positive efforts accordingly. Finally, it is necessary to discover different cases of online physical education, through the experiences of students and teachers (as well as their significance), in order to improve the universality of the experience and the associated courses, especially in online physical education classes [16].

## 6. Conclusions

The COVID-19 epidemic has shocked the world and affected all aspects of daily life. In a very short period of time, most educational institutions throughout the globe were forced to close, and face-to-face classes were replaced by online learning. This sudden change has led to huge pressures on educational institutions, but has also provided an opportunity for teachers and students to experience online learning. The epidemic has made us aware of the need to be fully prepared, in order to ensure that high-quality education continues in times of special conditions. So, as Bozkurt et al. [19] pointed out, the question should not be what we did during the COVID-19 epidemic, but how we will respond to the next impending disruption, in order not to repeat it. By classifying the most negative and positive characteristics of online classes, from the perspectives of students and teachers, the current research hopes to contribute to such preparation, emphasizing the challenges faced by physical education students taking online courses. The findings of the current study have plenty of implications: First of all, it is important to study the experience of online physical education in various countries, and compare and analyze the situation of online physical education in the global context. Therefore, it is necessary to review and structure the online physical education methods which disclose educational and cultural characteristics, and observe the effectiveness of online physical education in various countries. Secondly, it is necessary to highlight the potential of online physical education, which is linked to face-to-face physical education. Furthermore, according to the increase in professional knowledge obtained by physical education students and teachers through online physical education, it is essential to examine their respective efficiency and potential. Third, in order to achieve effective results, the classroom should follow Mayer’s learning model (as mentioned above). Finally, in the future, a richer theoretical framework of online physical education classes should be designed, in order to modify the existing teaching methods, content, evaluation, and other educational values, to teach online physical education classes more effectively to students. In addition, future research should also observe the efficiency and availability of various online platforms used by physical education students and teachers, as well as assessing their universality in official school sites, especially when developing new tools. However, this study had some limitations. First, the sample size was comparatively small and, as such, may not be representative of the total population. Finally, the homogeneous socio-cultural and religious characteristics of the selected samples may have led to bias. The researchers attempted to recognize any bias in the focus group discussions, in order to avoid such results. Taking into consideration the limitations of current study, the analysis of a larger sample could be implemented in future studies to obtain more representative results regarding the efficiency and availability of various online platforms used by physical education students and teachers.

## Figures and Tables

**Table 1:** Guidelines for focus group discussions.

1. Arrangements for Focus Group	2. Focus Group Discussions
Internet Laptop/computer/smart device Location Participants: Arrange time for meeting on Tencent Meeting Inform all participants about the time of meeting Login to Tencent Meeting 15 min before the focus group discussion time Research manual and participation agreement Interview/discussion guidelines Check the battery, and availability of electricity is confirmed Clock/Watch for time keeping Pen/notebook Extra laptop/smart device for taking notes Demographic characteristics document Recorder	At the Start Introduce researchers Explain the aims and objectives of the research Interview/discussion guidelines and precautions Ask participants to give consent for their participation Request for noise reduction At the End Thank the participants for their valuable time Ask participants if there was anything else that you want to share with the researchers Ask for availability of participants for future communication; for example, if another interview or queries were required Save the recordings

**Table 2:** Questions for focus group discussion.

Categories	Questions
Opening questions	Had you ever experienced online learning before the COVID-19 pandemic? Did you take online classes during the pandemic lockdown?
Exploratory questions	Could you please tell me about your experience with online learning? Was it good or bad? Please tell me what you liked about online learning, and what didn’t you like?
Transition questions	What differences did you experience between online learning and face-to-face learning at a university campus? What is your opinion, if online learning continues in the next academic year?
Key questions	Are you satisfied with online learning in physical education? Please explain your answers with reasons. What were your concerns during online learning? How did online learning affect your daily life? How did online learning affect your physical and mental health?
Closing questions	What are your suggestions for future online learning classes in physical education? Is there anything else that you would like to discuss with us?

**Table 3:** Description of the steps followed in the thematic analysis.

Steps	Description
1. Adjustment of the data and classification of student problems, accordingly	Recorded interviews/discussions, re-read copies, got main ideas, and distributed students, according to the challenges they faced.
2. Development of the preliminary codes, based on the objectives of current study	Defined thoughts according to the literature “poor performance, weight gain, and psychological issues faced by students due to online physical education classes during COVID-19”. Searched for examples in data and stated when the idea can be utilized.
3. Combination of the codes into main themes	Composed and categorized the information associated with each source code. Generated a visual representation to classify the code as a main topic or theme.
4. Modification of the themes	Analyzed the theme from two different levels. First, we checked whether they are related to code extraction. Second, we checked whether the theme matched the data.
5. Description of the themes	Each theme is analyzed and clearly defined. In addition, an informative name is selected for each theme (see the Material and Methods and Results sections).
6. Pre-setting the report	During manuscript preparation, we selected relevant citations representing good examples for each theme of the study.

**Table 4:** Background information of study participants (*n* = 56).

Characteristics	Frequency	Percentage
Gender		
Male	34	60.7
Female	22	39.3
Age		
<20	6	10.7
20–24	30	53.6
>24	20	35.7
Education Level		
Bachelor	21	37.6
Master	20	35.7
PhD	15	26.7

**Table 5:** Themes and sub-themes derived from focus group discussions.

Themes	Sub-Themes	Context	Examples
Knowledge of Online Learning	Familiarity with Online learning	Understanding of online learning, use of digital tools for learning	“I have experience of online learning in the past. So shifting from on-campus mode to online mode was easier for me.”
	Awareness of course content	Better learning environment	“I found online classes are better than on-campus classes because they helped me to focus more on learning.”
	Difficulties in course content understanding	Problems that students faced in understanding the contents	“I think online classes were very difficult because I did not understand the basic concepts. I did not feel easy during online lectures.”
Challenges Faced	Impact on performance in sport or physical activity	Online learning improved or worsened student performance in physical activities	“Physical education classes are different from other fields, because we have to perform different physical activities, which cannot be completed online. I found online physical education classes very difficult, and they badly impacted my performance.”
	Weight gain	Online learning has a negative impact on students’ health	“I tried my best to involve myself with physical activities through online classes, but I could not find it helpful. I have gained 10 kg weight in just three months.”
	Psychological issues	Positive or negative impact of online learning on students’ mental health	“Yes, I felt great stress during online learning classes. I had to spend three months at home. Physical activity helped me to release some stress, but stay-at-home made more stress.”
Suggestions for future online classes in physical education		Students’ suggestions for making online learning more interesting in future	“I think it would be better if teachers and students are well-prepared and well-trained to learn online again.”

## Data Availability

The data presented in this study are available on request from the corresponding author. The data are not publicly available due to confidential and research ethical issues.
